# Two cases of lysozyme-induced nephropathy with varying underlying diseases: myelodysplastic syndrome and chronic myelomonocytic leukemia

**DOI:** 10.1007/s13730-026-01170-x

**Published:** 2026-08-12

**Authors:** Hisashi Furukawa, Tsutomu Inoue, Minako Tanioka, Yuya Kusuyama, Mizuya Ito, Tatsuo Kondo, Daichi Fukaya, Hiroaki Amano, Koji Tomori, Yusuke Watanabe, Hiromichi Iwashita, Mei Hamada, Koji Okudera, Tomoiku Takaku, Yuichi Nakamura, Hirokazu Okada

**Affiliations:** 1 Department of Nephrology, Saitama Medical University, 38 Morohongo, Moroyama-machi, Iruma-gun, Saitama, 350-0495 Japan; 2 Department of Diagnostic Pathology, Saitama Medical University, Saitama, Japan; 3 Department of Hematology, Saitama Medical University, Saitama, Japan

**Keywords:** Lysozyme-induced nephropathy, Chronic myelomonocytic leukemia, Myelodysplastic syndrome

## Abstract

Lysozyme-induced nephropathy (LyN) is a rare kidney complication associated with chronic myelomonocytic leukemia (CMML). This condition is characterized by distinctive pathological features, including granules within proximal tubular cells that stain positively with periodic acid-Schiff or lysozyme staining, and granular deposits identified as high-electron-density deposits under electron microscopy. Although CMML is the most common cause of LyN, previous reports have suggested that its occurrence may be associated with the myelodysplastic syndrome (MDS). This report describes cases of LyN in a patient with MDS and CMML. Although both the LyN cases, caused by either MDS or CMML, exhibited similar kidney pathological findings, there was a clinical difference in the occurrence of peripheral blood monocytosis. The MDS-induced LyN showed no persistent peripheral blood monocytosis and, when the bone marrow biopsy specimens were stained with lysozyme, numerous positive cells were observed. Thus, hematologic disorders wherein the bone marrow comprises lysozyme-producing cells may induce LyN, even without peripheral blood monocytosis.

## Introduction

Kidney disorders frequently occur in hematological malignancies, and one-third of patients develop acute kidney injury (AKI) [1]. The primary causes of AKI in leukemia include kidney failure, tumor lysis syndrome, and drug-induced acute tubulointerstitial nephritis [2]. Chronic myelomonocytic leukemia (CMML) is associated with characteristic kidney pathologies, including monocyte infiltration of the kidneys and lysozyme-induced nephropathy (LyN) [3, 4]. In CMML, large quantities of lysozymes produced by monocyte-derived tumor cells are filtered through the glomeruli. Excessive lysozyme is reabsorbed by the proximal tubules, causing tubular damage and resulting in LyN [3, 5]. Although it is predominantly caused by CMML, LyN can occur in complications such as myelodysplastic syndrome (MDS) and acute myeloid leukemia [6]. To date, only a few cases of LyN-associated MDS have been reported. We report a rare case of MDS-associated LyN in comparison with a typical case of CMML-associated LyN.

### Case report

Case 1: An 80-year-old Japanese man presented with the chief complaint of lower limb edema. Eight years ago, he was diagnosed with hypertension and managed with amlodipine and irbesartan. He had been diagnosed with chronic kidney disease three years prior, with a serum creatinine level of approximately 1.2 mg/dL. One month earlier, a health checkup revealed progressive kidney dysfunction with a serum creatinine level of approximately 1.7 mg/dL. The patient visited our department five days earlier and was admitted for further evaluation of kidney impairment. He had a history of dyslipidemia, which was managed with pravastatin. Additionally, he also had a history of smoking 20 cigarettes/day for 20 years.

On admission, the physical examination findings were: height, 160.8 cm; weight, 55.6 kg; body temperature, 36.1 ℃; blood pressure, 144/63 mmHg; and heart rate, 65 beats/min. An examination of the head, neck, lungs, and heart revealed no abnormalities. The abdomen was flat and soft, with normal bowel peristalsis. The patient also exhibited lower limb edema. Chest radiography revealed a cardiothoracic ratio of 42.0%, with no pleural effusion or infiltrative shadows in the lung fields. A 12-lead electrocardiogram confirmed a normal sinus rhythm with a heart rate of 68 beats/min and the absence of ST changes. Non-contrast-enhanced abdominal computed tomography revealed normal kidney.

The laboratory data are presented in Table 1, indicating kidney dysfunction and elevated urine β2 microglobulin levels, which suggested a diagnosis of tubulointerstitial nephritis. He also presented with mild proteinuria. Complete blood cell counts revealed mild abnormalities, including leukopenia, anemia, and thrombocytopenia. Serum and urinary lysozyme levels were elevated. His serum lactate dehydrogenase level was normal, while the full blood Wilms' tumor 1 messenger ribonucleic acid (RNA) level was elevated at 1.1 × 10^2^ copies/μg RNA.

**Table 1:** Urine and blood test results for Case 1 and Case 2

	Case 1	Case 2	Reference value	Unit
*(Urinalysis)*				
SG	1.015	1.020	1.010–1.030	
pH	5.5	6	5.0–9.0	
Protein	1 +	1 +	–	
OB	1 +	3 +	–	
Glucose	–	–	–	
UPCR	0.55	3.64	< 0.15	g/gCr
NAG	25.1	14.8	0.0–8.0	IU/L
β2MG	14,754	10,740	0–250	μg/L
Lysozyme	750	1240	< 0.1	μg/mL
*(Urinary sediment)*				
RBC	1–4	20–29		/HPF
WBC	5–9	1–4		/HPF
Granular cast	5–9	5–9		/WF
*(Complete blood cell count)*				
WBC	3380	165,700	3300–8600	/μL
RBC	3.13	2.31	4.35–5.55	× 10^6^/μL
Hb	10.1	7.1	13.7–16.8	g/dL
Ht	31.6	22.9	40.7–50.1	%
PLT	9.1	12.5	15.8–34.8	× 10^4^/μL
*WBC differential*				
Neutro	56.30	70.70	42.4–75.0	%
Lympho	37.20	3.80	18.2–47.7	%
Mono	5.70	24.80	3.3–9.0	%
Eosino	0.00	0.00	0.4–8.6	%
Baso	0.80	0.70	0.2–1.4	%
blasts	Negative	Positive	Negative	
*(Serum chemistry)*				
TP	7.7	9.8	6.6–8.1	g/dL
ALB	4.2	4.1	4.1–5.1	g/dL
AST	21	40	13–30	U/L
ALT	17	19	10–42	U/L
LDH	214	479	124–222	U/L
Cr	1.47	3.8	0.65–1.07	mg/dL
eGFR	36.2	12.8		mL/min/1.73 m^2^
UA	8.1	9.0	3.7–7.8	mg/dL
UN	21.5	57.0	8.0–20.0	mg/dL
HbA1c	6.2	6.2	4.6–6.2	%NGSP
Na	142	137	138–145	mEq/L
Cl	109	104	101–108	mEq/L
K	4.1	4.9	3.6–4.8	mEq/L
Ca	9.0	9.1	8.8–10.1	mg/dL
IP	3.4	4.1	2.7–4.6	mg/dL
CRP	0.1	0.1	0–0.14	mg/dL
IgG	2065	3034	861–1747	mg/dL
IgA	210	1488	93–393	mg/dL
IgM	95	282	33–183	mg/dL
C3	82	47	73–138	mg/dL
C4	21	25	11–31	mg/dL
FLC κ/λ	1.79	0.77	0.26–1.65	
Lysozyme	109	325	5.0–10.2	μg/mL
ACE	10.6	20.1	7.0–25.0	U/L
*(Endocrine and tumor marker)*				
sIL-2R	679	1771	121–613	U/mL
WT1-mRNA	110	n.d	< 50	/μgRNA
*(Autoantibody)*				
ANA	Negative	Negative	Negative	
PR3-ANCA	Negative	Negative	Negative	
MPO-ANCA	Negative	Negative	Negative	
*(infection)*				
HBVAg	Negative	Negative	Negative	
HCVAb	Negative	Negative	Negative	
*(Immunoelectrophoresis)*				
BJP	Negative	Negative	negative	
IEP	Negative for M-protein	Negative for M-protein	negative	

SG, specific gravity; OB, occult blood; UPCR, urine protein/creatinine ratio; NAG, N-acetyilglucosamine; β2MG, β2-microglobulin; RBC, red blood cells; WBC, white blood cells; Hb, hemoglobin; Ht, hematocrit; PLT, platelet; Neutro, neutrophils; Lympo, lymphocytes; Mono, monocytes; Eosino, eosinophils; Baso, basophils; AST, aspartate aminotransferase; TP, total proteins; ALB, albumin; ALT, alanine aminotransferase; LDH, lactate dehydrogenase; Cr, creatinine; eGFR, estimated glomerular filtration rate; UA, uric acid; UN, urea nitrogen; HbA1c, hemoglobin A1c; Na, sodium; Cl, chloride; K, potassium; Ca, adjusted calcium; IP, inorganic phosphorus; CRP, c-reactive protein; Ig, immunoglobulin; C3, complement component 3; C4, complement component 4; FLC κ/λ, immunoglobulin free light chain κ/λ ratio; ACE, angiotensin-converting enzyme; sIL-2R, soluble interleukin 2 receptor; WT1-mRNA, Wilms' tumor 1 mRNA; ANA, antinuclear antibody; PR3-ANCA, proteinase3-anti-neutrophil cytoplasmic antibody; MPO-ANCA, myeloperoxidase-anti-neutrophil cytoplasmic antibody; HBVAg, hepatitis B surface antigen; HCVAb, hepatitis C virus antibody; BJP, urine Bence Jones protein; IEP, serum Immunoelectrophoresis; n.d., not done

**Fig. 1:**
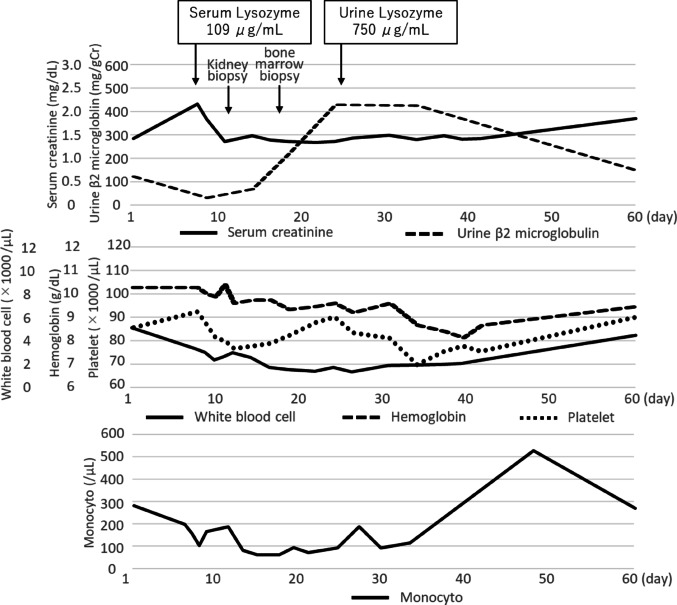
Clinical course of Case 1 Kidney biopsy was performed on day 11. A bone marrow biopsy was performed on day 15. Kidney function remained stable, with serum creatinine ranging from 1.4 to 1.8 mg/dL. Furthermore, pancytopenia, which was mild at admission, worsened over time

**Fig. 2:**
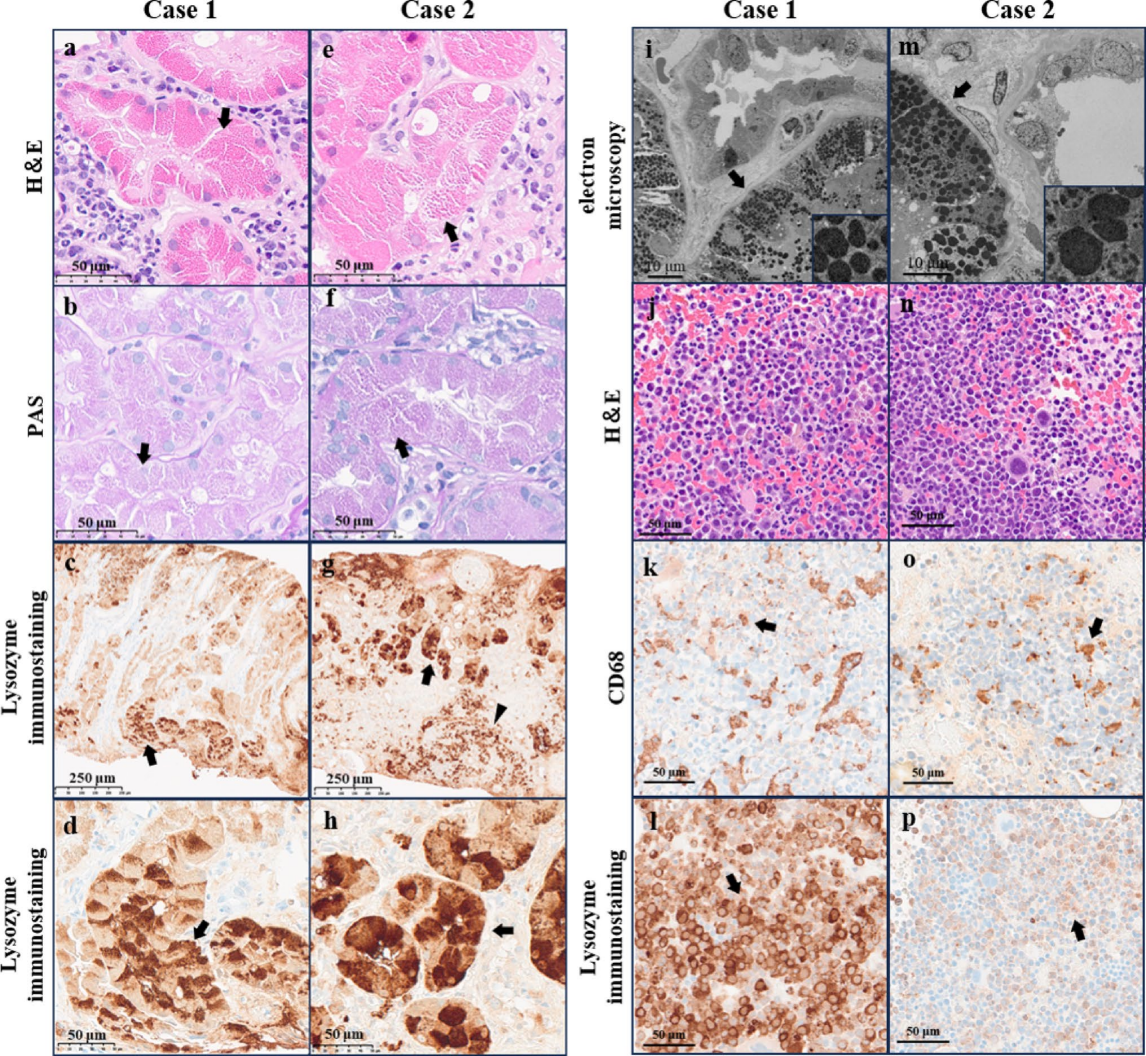
Findings from kidney and bone marrow biopsy specimens **a** hematoxylin and eosin staining (H&E) of kidney biopsy in case 1. **b** Periodic acid-Schiff staining (PAS) of kidney biopsy in case 1 **c** Lysozyme immunostaining of kidney biopsy in case 1. **d** Lysozyme immunostaining of kidney biopsy in case 1. **e** H&E of kidney biopsy in case 2. f, PAS of kidney biopsy in case 2. **g** Lysozyme immunostaining of kidney biopsy in case 2. **h** Lysozyme immunostaining of kidney biopsy in case 2. **i** electron microscopy of kidney biopsy in case 1. **j** H&E of bone marrow biopsy in case 1. **k** CD68 immunostaining of bone marrow biopsy in case 1. l, Lysozyme immunostaining of bone marrow biopsy in case 1. m, electron microscopy of kidney biopsy in case 2. **n** H&E of bone marrow biopsy in case 2. **o** CD68 immunostaining of bone marrow biopsy in case 2. p, Lysozyme immunostaining of bone marrow biopsy in case 2. In kidney biopsy, H&E staining revealed eosinophilic inclusions within the cytoplasm of the proximal tubules(arrow) in both cases 1 and 2 (**a**, **e**). Cytoplasmic granules witthin the proximal tubules (arrow) are weakly positive for PAS (**b**, **f**). There were no significant differences in the shape or staining characteristics of the granules observed with H&E or PAS staining. Lysozyme immunostaining revealed the presence of cytoplasmic lysozymes (arrow) in the proximal tubules (**c**, **d**, **g**, **h**). Compared with case 2, the proximal tubules positive for lysozyme staining showed a more focal distribution in case 1. The staining pattern within a single proximal tubule was more marbled in case 1 than in case 2. Case 2 shows interstitial infiltration of lysozyme-positive cells (triangle in **g**). Electron microscopy reveals granular high-electron-density deposits (arrow) in the proximal tubules (inset demonstrates higher magnification) (**i**, **m**). There was no significant difference in the degree of deposition between cases 1 and 2. No crystalline, fibrous, or tubular structures were observed. In the bone marrow biopsy, both cases 1 and 2 showed an increase in cell numbers, including monocytes (**j**, **n**). Immunostaining of the bone marrow biopsy revealed CD68-positive and lysozyme-positive cells (arrow) in cases 1 and 2 (**k**, **l**, **o**, **p**). The proportion of CD68-positive cells was lower than that of lysozyme-positive cells

The clinical course of the patient is shown in Fig. 1. Kidney biopsy was performed. The kidney biopsy results are shown in Fig. 2. Light microscopy revealed nine glomeruli, none of which were globally sclerosed. The infiltration of various inflammatory cells and mild fibrosis were observed in the interstitium. Glomerular changes were minor, except for a slight proliferation of the mesangium in some glomeruli. Diffuse intracytoplasmic inclusions were observed in the proximal tubules (Fig. 2a, b). Immunofluorescent staining for IgG, IgA, IgM, C1q, C3c, and C4c revealed no significant positivity. Immunohistochemical examination revealed lysozyme-positive intracytoplasmic inclusions in the proximal tubules (Fig. 2c, d). Electron microscopy revealed numerous deposits with high electron density in the cytoplasm of the proximal tubules (Fig. 2i). Based on pathological findings, the patient was diagnosed with LyN, prompting the evaluation of an underlying hematologic disease involving monocytosis. Therefore, a bone marrow biopsy was performed. Various myeloid cells, including monocytes, were observed in the bone marrow biopsy specimens (Fig. 2j). The marrow was hypercellular, with an increased number of megakaryocytes and no obvious dysplastic changes in megakaryocytes or erythrocytes were observed. In the bone marrow smear preparation, the myeloid/erythroid ratio was 9.92, indicating a decrease in the erythroid ratio, and granulocytes exhibited chromatin condensation abnormalities and degranulation. The myeloblast ratio was 0.8%. Moreover, monocyte count increased by 14.8%. Immunohistochemical staining revealed an increase in CD68-positive cells and an even greater increase in lysozyme-positive cells (Fig. 2k, l). A diagnosis of MDS with a low blast count was established based on the bone marrow biopsy results and the clinical course of progressive pancytopenia. After consultation with the hematology department, chemotherapy was considered, but deferred because of the high risk of cytopenia, and the patient was monitored without treatment. During the observation period, peripheral blood monocytes increased to 521/μL; however, absolute monocytosis was not sustained.

Case 2: An 81-year-old Japanese man presented with mild lower limb edema. Three years previously, he had been diagnosed with chronic kidney disease (serum creatinine level ~ 1.2 mg/dL). He visited the hematology department of our hospital two years ago because of leukocytosis and was diagnosed with CMML. No evidence of acute transformation was observed; chemotherapy was deferred, and the patient remained under observation. Thereafter, his serum creatinine level stabilized at approximately 1.7 mg/dL. He was diagnosed with idiopathic uveitis one year prior and treated with glucocorticoid eye drops in the ophthalmology department, which led to improvement. Kidney dysfunction had progressively deteriorated over the previous 11 months. He presented to our department two months before his serum creatinine level rose to 3.0 mg/dL. One month earlier, he had undergone a kidney biopsy, which revealed tubulointerstitial nephritis. The patient was admitted to our department for the management of tubulointerstitial nephritis. He had a history of well-controlled diabetes mellitus that was managed using linagliptin. Moreover, he had smoked 10 cigarettes per day for 20 years.

On admission, the physical examination findings were: height, 158.0 cm; weight, 47.8 kg; body temperature, 37.1℃; blood pressure, 149/100 mmHg; and heart rate, 69 beats/min. Examination revealed no abnormalities in the head and neck, including in alveolar breath or heart sounds. The abdomen was flat and soft, with normal bowel peristalsis. However, the patient exhibited mild lower limb edema. Chest radiography revealed a cardiothoracic ratio of 49.7%, with no pleural effusion or infiltrative shadows in the lung fields. A 12-lead electrocardiogram demonstrated a normal sinus rhythm with a heart rate of 87 beats/min and no ST changes. Non-contrast-enhanced abdominal computed tomography revealed normal kidney.

**Fig. 3:**
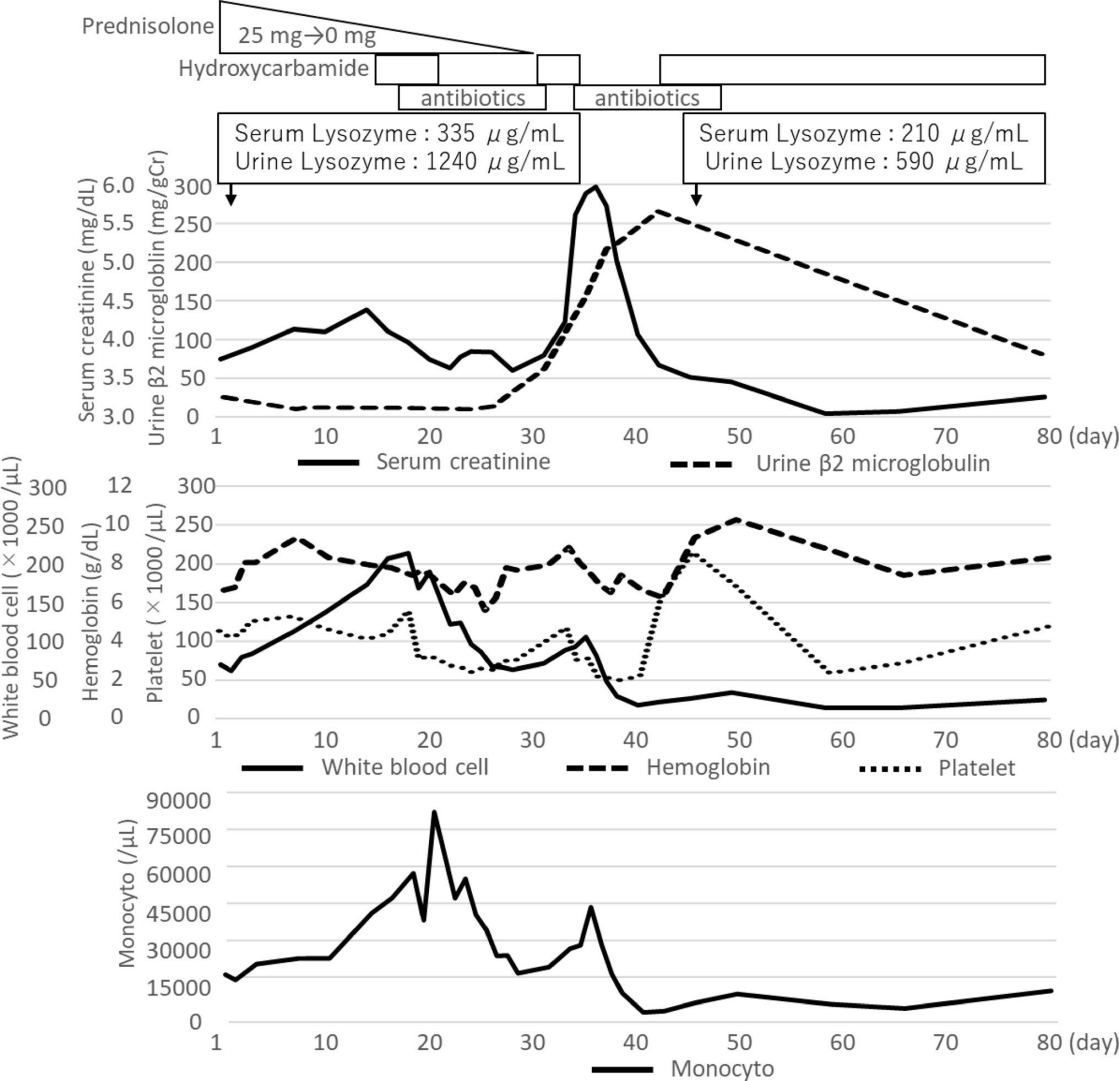
Clinical course of Case 2. Prednisolone was initiated on day one, but as the underlying disease was identified as lysozyme-induced nephropathy, the dose was tapered off over one month. Hydroxycarbamide was administered on day 16. A urinary tract infection developed on day twenty-three, leading to discontinuation of hydroxycarbamide and treatment with imipenem and cilastatin. The fever resolved within a few days, and hydroxycarbamide treatment was restarted on day thirty-two. On day thirty-five, urinary tract infection recurred; therefore, hydroxycarbamide was discontinued, and treatment with meropenem was initiated. With improvement in the urinary tract infection, hydroxycarbamide was restarted on day forty-four. Following treatment, the serum creatinine, lysozyme, and urinary lysozyme levels decreased

The laboratory data are presented in Table 1, indicating kidney dysfunction and elevated urine β2 microglobulin levels, indicating tubulointerstitial nephritis. The patient exhibited severe proteinuria; however, his serum albumin levels were normal. A complete blood cell count revealed marked leukocytosis, predominantly involving neutrophils and monocytes. Serum and urinary lysozyme levels were elevated. The patient’s clinical course is shown in Fig. 3. Tubulointerstitial nephritis and uveitis syndrome (TINU) was initially suspected, and glucocorticoid therapy was initiated; however, his kidney dysfunction deteriorated. Consequently, the pathological findings were reevaluated. Pathological findings are shown in Fig. 2. Light microscopy revealed 40 glomeruli, two of which were globally sclerosed. The tubulointerstitium exhibited a diffuse infiltration of various inflammatory cells. Diffuse and abundant eosinophilic inclusions within the cytoplasm of the proximal tubules (Fig. 2e) and focal interstitial fibrosis were observed, which were weakly positive for PAS (Fig. 2f), fuchsine-positive for Masson trichrome, and negative for PAM. Although some glomeruli exhibited mild proliferative changes in the mesangium and a slight double contour, other glomerular changes were minimal. Immunofluorescence examination revealed partial positivity for IgA and C3c, whereas it was negative for IgG, IgM, C1q, C4c, κ, and λ light chains. Immunohistochemical examination revealed myeloperoxidase- and CD68-positive cells in a portion of the interstitium. Furthermore, Immunohistochemical examination also demonstrated that the cytoplasmic granular inclusions in the proximal tubule and a portion of the interstitium were diffusely positive for lysozyme (Fig. 2g, h). Electron microscopy revealed abundant intracytoplasmic inclusions with high electron density (Fig. 2m). Based on these pathological findings, the diagnosis was revised to LyN, and glucocorticoids were discontinued. The findings from the bone marrow biopsy performed two years ago were consistent with CMML. Various myeloid cells were observed in the bone marrow biopsy specimens, and a significant increase in monocytes was noted (Fig. 2j). While the marrow was hypercellular with separated multilobed nuclei in the megakaryocytes, no obvious dysplastic changes in erythrocytes were observed. In the bone marrow smear preparation, the myeloid/erythroid ratio was 10.83. Granulocytes exhibited degranulation, and the myeloblast ratio was 2.1%. The mature monocyte fraction was 22.6%, and the immature monocyte fraction was 5.6%. Immunohistochemical staining revealed an increase in CD68-positive cells and lysozyme-positive cells (Fig. 2o, p). Based on the diagnosis of LyN, Hydroxycarbamide chemotherapy was initiated for CMML. Hydroxycarbamide was temporarily discontinued, and kidney dysfunction was exacerbated owing to urinary tract infection. However, the continuation of hydroxycarbamide therapy halted further kidney deterioration, with serum creatinine levels stabilizing at approximately 3.3 mg/dL. Additionally, chemotherapy reduces urinary and serum lysozyme levels.

## Discussion

Herein, we report two cases of LyN with different underlying diseases: MDS and CMML. LyN is a rare condition that occurs frequently in association with CMML. Lysozymes are enzymes synthesized by monocytes and are produced in large quantities by monocyte-derived tumor cells in CMML [5]. Lysozyme is a small protein (15 kDa) freely filtered through the glomerulus [7]. The filtered lysozyme is reabsorbed by the proximal tubule, endocytosed, and degraded within the phagolysosomes [8]. Excessive lysozyme uptake beyond this threshold damages proximal tubules, resulting in LyN [9].

CMML often has LyN as an underlying disease; however, it has also been suggested to be caused by MDS (14% of LyN) [6]. Case 2 presented with a typical course of LyN with CMML as its etiology. However, at the time of admission, case 1 had no clear hematological disorder. Although the clinical findings strongly suggested lysozyme-induced nephropathy, which induced consideration of comorbid CMML, according to the diagnostic criteria in the 5th Edition of the World Health Organization Classification of Hematolymphoid Tumors (WHO Classification) [10], the absence of persistent absolute monocytosis or an increased monocyte fraction in the peripheral blood ruled out CMML. Based on a 0.8% blast percentage, the absence of findings that were suggestive of other hematologic malignancies, the absence of sustained monocytosis, and the presence of granulocyte degranulation and abnormal chromatin aggregation, we diagnosed the patient with MDS in accordance with the WHO classification criteria. In Case 1, as no condition other than MDS explained the elevated serum lysozyme levels, LyN was considered. Peripheral blood monocytosis and elevated lysozyme levels are uncommon and are rarely reported in patients with MDS. However, studies in Japan have reported that serum lysozyme levels are elevated in patients with MDS compared to healthy individuals [11]. Furthermore, a multicenter study examining LyN reported elevated serum and urine lysozyme levels in patients with LyN associated with MDS [6]. We hypothesized that a small group of patients with MDS and elevated lysozyme levels might have LyN. We performed lysozyme staining of bone marrow biopsy specimens from both cases 1 and 2 (Fig. 2l, p). In CMML-induced LyN (case 2), the presence of a large number of lysozyme-positive cells was expected owing to the increase in the absolute monocyte count. However, even in MDS-induced LyN (case 1), without a persistent monocyte increase, a large number of lysozyme-positive cells were similarly present in the bone marrow. The findings in case 1 suggest that even in MDS cases with normal peripheral blood monocyte counts, a subgroup of patients have lysozyme-producing cells within the bone marrow. We also performed CD68 staining of Case 1 and Case 2 samples (Fig. 2k, o). Although CD68-positive cells were present, their proportion was lower than that of lysozyme-positive cells. This finding was particularly apparent in the MDS case(Case 1), suggesting that various myeloid cells, including monocytes, may produce lysozyme in MDS. These patients may develop LyN following an increase in serum lysozyme levels even in the absence of increased peripheral blood monocytes or a marked increase in bone marrow monocytes. This finding from a single case is hypothesis-generating, but warrants validation in studies with a large sample and further research on bone marrow serum lysozyme levels and lysozyme-producing cells in patients with MDS.

The pathological findings of LyN often involve cellular infiltration, including eosinophils, around the proximal tubules. A hallmark feature is the deposition of eosinophilic, PAS-positive granular material within the proximal tubules. Furthermore, granular or mottled lysozyme staining within the proximal tubule cells is also a characteristic finding. Electron microscopy has demonstrated granular deposits with high electron density [6]. Case 1 and 2 exhibited similar pathological findings and were diagnosed with LyN. The pathological findings of the LyNs were characteristic. When PAS staining revealed granular deposits in the proximal tubules, lysozyme staining, electron microscopy, and measurements of serum and urinary lysozyme levels were performed. It is believed that this additional testing can not only identify the cause of the tubulointerstitial disorder but also facilitate the diagnosis of an underlying hematologic disorder associated with monocytosis. A comparison of cases 1 and 2 revealed highly consistent findings from light microscopy and electron microscopy. LyN may present with similar histopathological features despite a different underlying disease. However, when comparing the two cases, case 1 showed weaker, less widespread lysozyme staining. Case 1 has lower serum and urinary lysozyme levels than case 2. In LyN, serum and urinary lysozyme levels may not only aid diagnosis but also indicate disease activity and the extent of tubular damage. Further research is needed to compare serum and urinary lysozyme levels with the degree of tubular damage in LyN. Additionally, in Case 2, a small number of cells positive for CD68, MPO, and lysozyme were observed in the tubulointerstitium. This finding suggests a direct infiltration by leukemia cells. Several reports have described AKI caused by leukemic cell infiltration in patients with CMML. Previous reports noted diffuse leukemic cell infiltration [4, 12]. Conversely, Case 2 exhibited a low number of infiltrating leukemic cells, suggesting that LyN was the primary cause of AKI.

Compared to Case 1, Case 2 presented with more severe kidney dysfunction. Case 2 showed a greater number of damaged proximal tubules in lysozyme stained tissues, which may have contributed to kidney dysfunction. Although leukemic cell infiltration is not the primary pathology of AKI, it may also impact kidney function. Case 2 revealed more globally sclerosed glomeruli and interstitial fibrosis than Case 1. The interval from initial diagnosis of kidney dysfunction prior to hospitalization to onset of LyN was also longer in Case 2. It is possible that Case 2 had developed more advanced chronic kidney disease prior to onset of LyN. The differences in severity of kidney damage between the two cases are caused by various factors. These differences are likely due to conditions unique to the individual cases rather than the underlying disease itself.

LyN clinical findings included elevated serum and urinary lysozyme levels in all cases. Urinalysis reported proteinuria in 77% of cases [6]. Proteinuria was observed in Cases 1 and 2. In patients with LyN, lysozymes and tubular proteinuria have been reported as non-albumin urinary protein [6]. A discrepancy between the qualitative and quantitative urine protein tests suggested that the proteinuria was not solely albuminuria but also included urinary lysozyme and tubular proteinuria. However, quantitative testing for albuminuria was not performed in these cases. Quantitative assessment of albuminuria and proteinuria is important to induce suspicion of the presence of urinary lysozyme.

In conclusion, nephrologists must be familiar with the characteristics of LyN, as it can occur in various hematologic disorders characterized by monocytosis or elevated lysozyme levels. Kidney diseases are diagnosed when they originate from hematological disorders that involve monocytosis or elevated lysozyme levels. Identifying the cause of kidney disease can aid in the diagnosis of the underlying hematological disorders. Furthermore, even in patients with MDS and normal peripheral blood monocyte counts, LyN may occur when there is an increase in bone marrow lysozyme-producing cells.
